# Dextran-Curcumin Nanosystems Inhibit Cell Growth and Migration Regulating the Epithelial to Mesenchymal Transition in Prostate Cancer Cells

**DOI:** 10.3390/ijms22137013

**Published:** 2021-06-29

**Authors:** Emilia Bevacqua, Manuela Curcio, Federica Saletta, Orazio Vittorio, Giuseppe Cirillo, Paola Tucci

**Affiliations:** 1 Department of Pharmacy, Health and Nutritional Sciences, University of Calabria, 87036 Rende, Italy; emilia.bevacqua@unical.it (E.B.); manuela.curcio@unical.it (M.C.); giuseppe.cirillo@unical.it (G.C.); 2 Lowy Cancer Research Centre, Children’s Cancer Institute, University of New South Wales, High Street, Randwick, NSW 2052, Australia; FSaletta@ccia.org.au (F.S.); OVittorio@ccia.unsw.edu.au (O.V.); 3 School of Women’s and Children’s Health, University of New South Wales, Kensington, NSW 2052, Australia; 4 ARC Centre of Excellence for Convergent BioNano Science and Technology, Australian Centre for NanoMedicine, University of New South Wales, Kensington, NSW 2052, Australia

**Keywords:** prostate cancer, self-assembling nanoparticles, combination therapy, curcumin, epithelial to mesenchymal transition

## Abstract

Functional nanocarriers which are able to simultaneously vectorize drugs to the site of interest and exert their own cytotoxic activity represent a significant breakthrough in the search for effective anticancer strategies with fewer side effects than conventional chemotherapeutics. Here, we propose previously developed, self-assembling dextran-curcumin nanoparticles for the treatment of prostate cancer in combination therapy with Doxorubicin (DOXO). Biological effectiveness was investigated by evaluating the cell viability in either cancer and normal cells, reactive oxygen species (ROS) production, apoptotic effect, interference with the cell cycle, and the ability to inhibit cell migration and reverse the epithelial to mesenchymal transition (EMT). The results proved a significant enhancement of curcumin efficiency upon immobilization in nanoparticles: IC_50_ reduced by a half, induction of apoptotic effect, and improved ROS production (from 67 to 134%) at low concentrations. Nanoparticles guaranteed a pH-dependent DOXO release, with a more efficient release in acidic environments. Finally, a synergistic effect between nanoparticles and Doxorubicin was demonstrated, with the free curcumin showing additive activity. Although in vivo studies are required to support the findings of this study, these preliminary in vitro data can be considered a proof of principle for the design of an effective therapy for prostate cancer treatment.

## 1. Introduction

Prostate cancer (PCa) is the most common malignancy and the second most common cancer-related cause of mortality in men [1,2]. Commonly, treatment for PCa involves surgery, radiotherapy and chemotherapy [3,4], with the latter being the main approach for the treatment of metastatic tumors [5,6]. Among others, Doxorubicin (DOXO), an anthracycline antibiotic causing DNA damage and producing radical species, is widely used as a cytotoxic agent for the treatment of prostate cancers [7,8]. However, DOXO is known to be associated with serious side effects such as bone marrow ablation, immunosuppression and cardiomyopathy, dramatically limiting its dosage regimen [9,10,11]. Moreover, since DOXO treatment can induce multidrug resistance, leading to treatment failure upon disease recurrence [12,13,14,15], there is an urgent need for targeted and less toxic therapies to treat PCa which would improve patient overall survival as well as the quality of life of long-term survivors [16,17]. Several strategies have been proposed to reduce DOXO systemic toxicity and improve the therapeutic efficacy, including combination therapy with naturally occurring compounds showing high anticancer efficacy, low cross-toxicity to normal tissues, and the ability to synergize the antineoplastic effects of conventional chemotherapeutics [18,19,20].

Polyphenols, effecting different biological pathways simultaneously, have been shown to address these requirements, although their translation to clinical practice suffers from some limitations related to either their low chemical stability or unfavorable pharmacokinetic profiles [21,22,23,24]. In order to deliver polyphenols to cancer cells more efficiently, different formulations have been proposed in the literature, including nanoparticle systems and polymer conjugates [25,26,27,28]. The conjugation of polyphenols to water-soluble polymers enhances their ease of administration and solubility in biological fluids [29,30] and, more importantly, guarantees therapeutic efficacy by improving their pharmacokinetic profile [31,32] and overcoming cellular resistance mechanisms [33]. The conjugation of polysaccharides and proteins to polyphenols allows the synthetic strategy to be finely tuned in order to address the requirements of high biocompatibility and efficiency, and to avoid the formation of undesirable reaction byproducts [34,35,36].

We previously demonstrated that the use of solid biocatalysts, consisting of immobilized laccase, was an effective approach for the conjugation of polyphenolic compounds (e.g., catechin and curcumin) to either proteins (e.g., gelatin and albumin) or polysaccharides (e.g., dextran and alginate) [37]. Such bioconjugates displayed both improved antioxidant and anticancer activity [38,39]. Moreover, we showed that lipophilic molecules such as curcumin conferred amphiphilic characteristics to high molecular weight dextran, resulting in self-assembly dextran/curcumin (DEX/CUR) conjugates. Such nanoparticles were successfully used to vectorize methotrexate to breast cancer cells, thereby synergizing its cytotoxic activity [40].

Here, we extend this approach using DEX/CUR nanoparticles to vectorize DOXO into PC-3 cancer cells, investigating the molecular mechanisms exerting antitumoral effects with particular regard to cell proliferation inhibition and the epithelial-to-mesenchymal transition (EMT) pathway. To better assess these findings, control experiments were conducted comparing the effect of DEX/CUR conjugates on cell treatment with that of free Curcumin (CUR) combined with DOXO.

## 2. Results

### 2.1. Biological Characterization of DEX/CUR Conjugate

**Figure 1:**
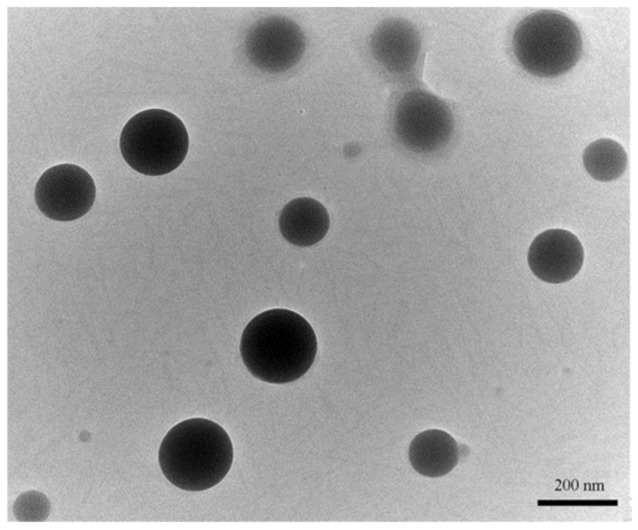
TEM images of DEX/CUR (scale bar 200 nm) showing microspheres with an average diameter of 290 nm. Scale bar 200 nm. Reproduced from [40]. MDPI (2020).

As previously demonstrated, the coupling of CUR to Dextran (DEX) via Laccase catalysis allowed us to obtain a DEX/CUR conjugate which is capable of self-assembly into spherical nanoparticles with a mean diameter of 290 nm (Figure 1) and a Polydispersity Index (PDI) of 0.21, as determined by combined dynamic light scattering (DLS) and transmission electron microscopy (TEM) analyses [40].

**Figure 2:**
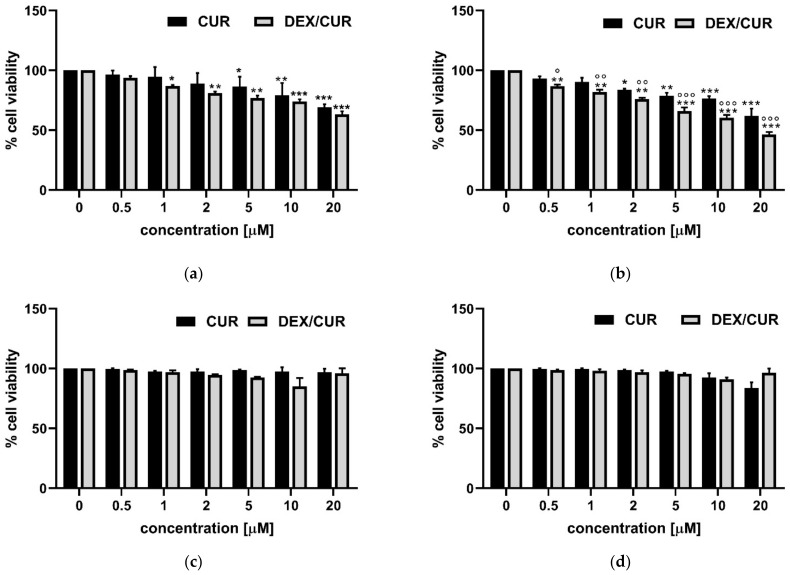
(**a**,**b**) PC-3 and (**c**,**d**) PNT-2 viability after (**a**,**c**) 24 h and (**b**,**d**) 48 h treatment with CUR (black bars) and DEX/CUR (grey bars). Data were reported as mean values ± SD of three independent experiments. Two-way ANOVA followed by Dunnett’s method: * *p* < 0.01, ** *p* < 0.001, *** *p* < 0.0001 vs. control. ° *p* < 0.01, °° *p* < 0.001, °°° *p* < 0.0001 vs. CUR equivalent concentration.

To validate the biological activity of such nanoparticles, we firstly evaluated the effect of free CUR on human prostate cancer cells (PC-3), obtaining the expected concentration- and time- dependent cell viability [41,42]. In particular, cell viability decreased with increasing CUR concentration with a similar trend at 24 and 48 h (Figure 2a,b), until reaching a viability of almost 60% after 48 h treatment with 20 μM CUR.

Then, PC-3 cells were treated with increasing concentrations of the DEX/CUR conjugate for 24 and 48 h, showing that the improved stability of the polyphenols upon conjugation resulted in an increased anticancer activity, with a viability reduced to 45% after 48 h treatment with 20 μM CUR equivalent concentration.

More importantly, such enhancement in the cytotoxic effect was observed specifically in cancer cells, while the viability of healthy prostate PNT-2 cells was not significantly affected by either free or conjugated CUR in the tested concentration range after 24 or 48 h treatment (Figure 2c,d).

**Figure 3:**
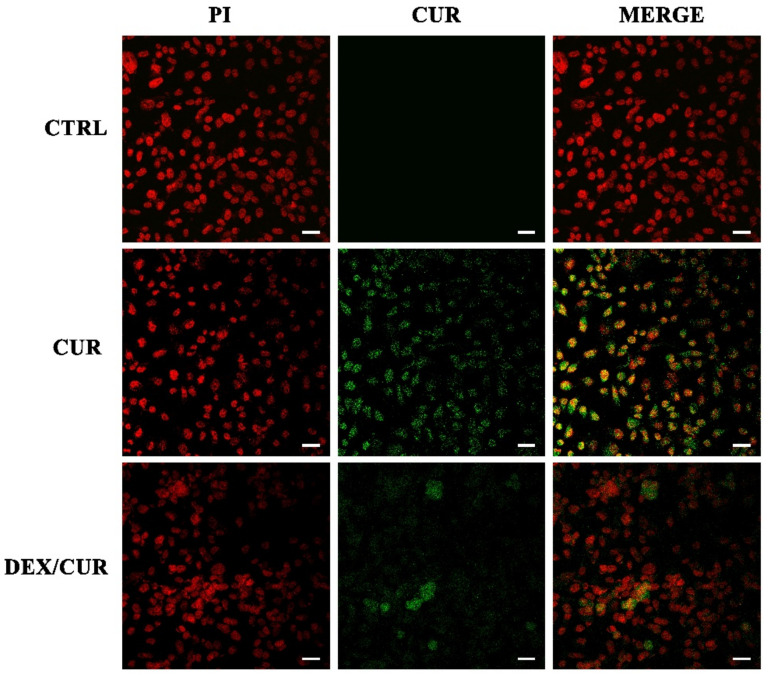
Confocal microscopy images (scale bar 12.5 μm, magnification 400×) of PC-3 cells after 4 h treatment with CUR or DEX/CUR at 20 µM CUR equivalent concentration. Red and green fluorescence correspond to cell nuclei and CUR or DEX/CUR, respectively.

Immunofluorescence experiments (Figure 3) demonstrated that after 4 h incubation, the green fluorescence signal of CUR was present within cells treated with both CUR and DEX/CUR.

**Figure 4:**
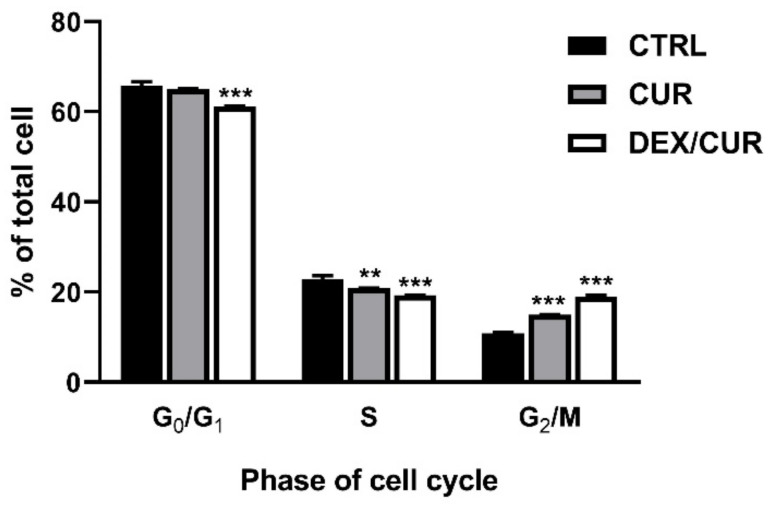
Cycle analysis by flow cytometry of PC-3 cells treated with CUR and DEX/CUR for 24 h at 20 µM CUR equivalent concentration. Data were reported as mean values ± SD of three independent experiments. Two-way ANOVA followed by Dunnett’s method: ** *p* < 0.001, *** *p* < 0.0001 vs. control.

According to the cell cycle analysis (Figure 4), both CUR and DEX/CUR increased the 4N DNA (G2/M phase) cell fraction indicating a G2/M phase arrest, but decreased the cell fraction in the G1 or S phase, particularly upon DEX/CUR treatment.

**Figure 5:**
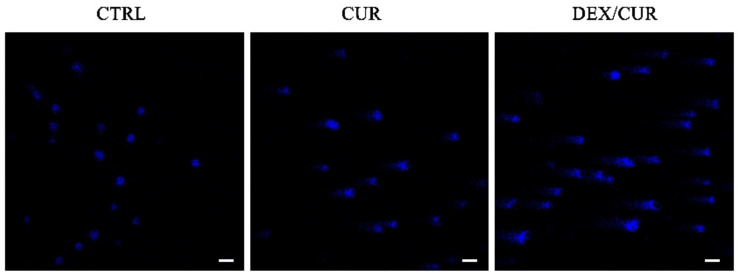
Fluorescent microscope images (scale bar 25 μm, magnification 200×) of PC-3 DNA damage analyzed by Comet assay after 36 h treatment with CUR and DEX/CUR at 20 µM CUR equivalent concentration.

To verify whether the decrease in cell growth was associated with apoptosis, Comet assay was performed, with the results depicted in Figure 5. Control cells showed round nuclei without comet tails, which instead were clearly evident after treatment with CUR or, even more, with DEX/CUR, indicating a high apoptotic effect for both treatments.

To investigate the level of reactive oxygen species (ROS) induced by CUR and DEX/CUR conjugates (CUR equivalent concentration of 5, 10, and 20 μM) in both normal and cancer prostate cell lines, we used CM-H2DCFDA, a cell-permeable fluorogenic probe which is deacetylated by intracellular esterases and oxidized in the presence of ROS, becoming highly fluorescent (Figure 5).

**Figure 6:**
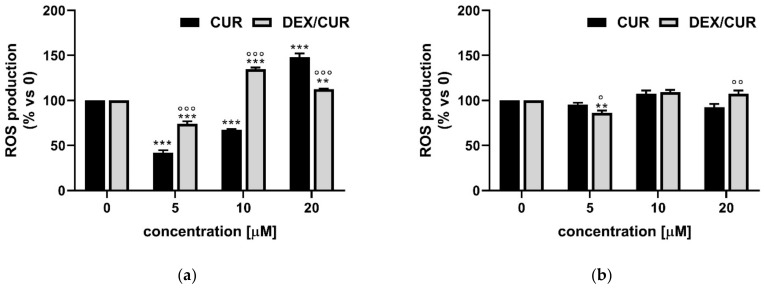
ROS levels (expressed as % of ROS production compared to the control) determined by FACS analysis using the CM-H2DCFDA probe in (**a**) PC-3 and (**b**) PNT-2 cells after 48 h treatment with CUR and DEX/CUR (5, 10, and 20 CUR equivalent concentration). Data were reported as mean values ± SD of three independent experiments. Two-way ANOVA followed by Dunnett’s method: ** *p* < 0.001, *** *p* < 0.0001 vs. control. ° *p* < 0.01, °° *p* < 0.001, °°° *p* < 0.0001 vs. CUR equivalent concentration.

The results demonstrated a concentration-dependent effect on ROS production in PC-3 cells (Figure 6a) where increased CUR concentration induced a significant increase in ROS. Different behavior was observed in cells treated with DEX/CUR. At low CUR equivalent concentrations (5 and 10 µM), an increase in ROS production was recorded in conjugate treated PC-3 cells, becoming higher than the control at 10 µM (134 vs. 67%). Importantly, these data are consistent with those obtained in the viability assay (Figure 2). A further increase in CUR equivalent concentrations (20 µM) resulted in an enhancement of the ROS production for free CUR up to 147%. Notably, at this concentration, the different chemical nature of the conjugate vs. the native polyphenol was responsible for a slight reduction of detected ROS in cells treated with 20 µM conjugate (134%) compared to 10 µM treatment (112%).

Interestingly, the basal level of ROS production in normal PNT-2 cells was not affected by either CUR or DEX/CUR, with negligible differences in control and treated cells (Figure 6b).

**Figure 7:**
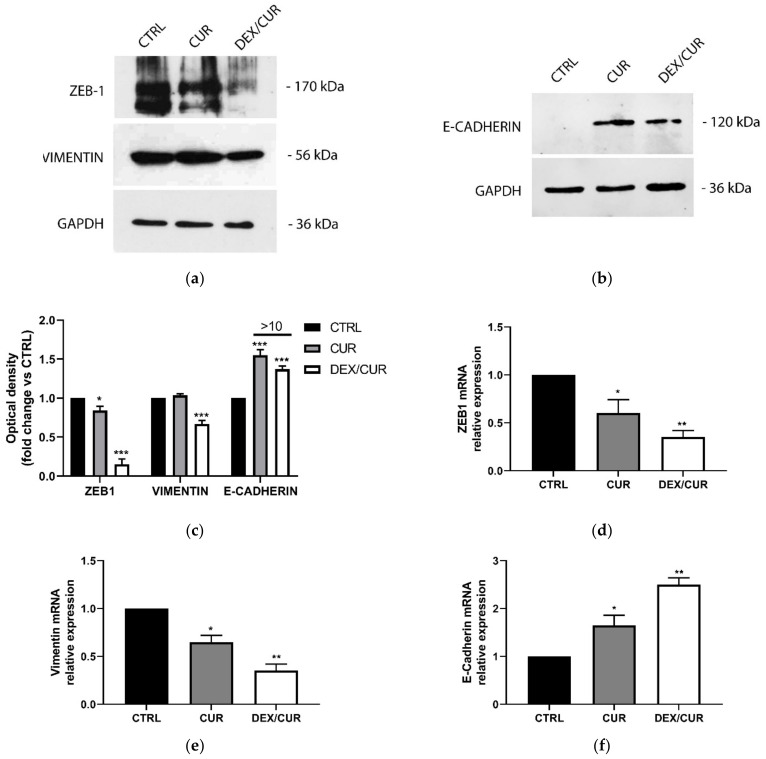
(**a**,**b**) Western blot, (**c**) Western blot quantitative densitometry analysis, and (**d**–**f**) qRT-PCR evaluating the mRNA levels of (**a**,**d**) ZEB-1, (**a**,**e**) Vimentin, and (**b**,**f**) E-cadherin after 48 h treatment with CUR and DEX/CUR at 20 µM CUR equivalent concentration. GAPDH levels were used as control protein for WB and as internal control gene for qRT-PCR. Data were reported as mean values ± SD of three independent experiments. (**d**–**f**) one-way and (**c**) two-way ANOVA followed by Dunnett’s method: * *p* < 0.01, ** *p* < 0.001, *** *p* < 0.0001 vs. control.

Western blot and qRT-PCR analyses were performed after incubation of PC-3 cells with both CUR and DEX/CUR to demonstrate their role in the regulation of tumorigenesis and tumor progression markers (Figure 7). Following treatment of PC-3 cells with 20 μM CUR equivalent, western blot and qRT-PCR analysis demonstrated a significant reduction in ZEB-1 (Figure 7a,d) at both the mRNA and protein levels. In particular, the transcription was reduced by 40% compared to the control, while the protein level was 15%, as demonstrated by quantitative densitometry analysis. Consequently, the treatment induced the reduction of vimentin (Figure 7a,e) and increase of E-cadherin (Figure 7b,f) expression. Even more consistent results were obtained for cells treated with DEX-CUR, suggesting an involvement in the epithelial-mesenchymal transition (EMT) of cancer cells, since ZEB-1 protein is one of the major regulators of EMT acting by two main pathways, namely, the down-regulation of epithelial proteins such as E-cadherins, and the over-expression of mesenchymal markers such as vimentin [43].

**Figure 8:**
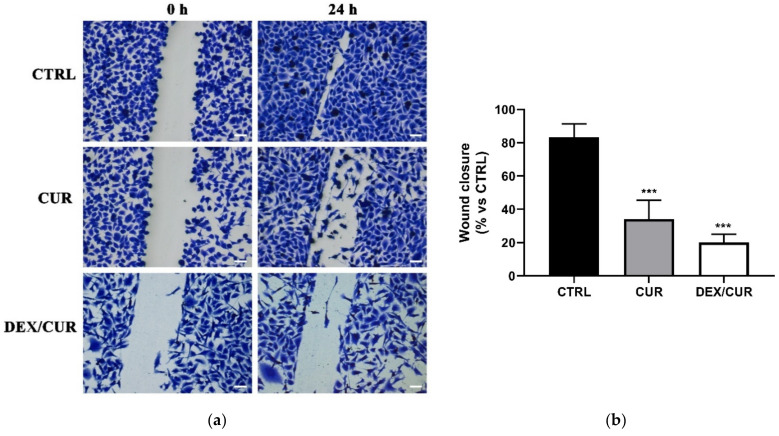
(**a**) Wound healing assays of PC-3 cells (scale bar 50 μm, magnification 100×) treated with CUR or DEX/CUR (20 µM CUR equivalent concentration). Monolayers were scratch wounded with a p200 tip. The wound closure is illustrated by showing the wound immediately (0 h) and 24 h after the scratch. (**b**) Quantitative densitometry analysis of wound closure. Data were reported as mean values ± SD of three independent experiments. One-way ANOVA followed by Dunnett’s method: *** *p* < 0.0001 vs. control.

To determine the consequent changes in metastasis ability, a wound healing assay was performed. The results (Figure 8) showed that CUR moieties were effective in inhibiting cell migration, with the scratch wound of cells failing to close compared to the control (Figure 8a,b).

### 2.2. Curcumin Nanoparticles Increases Sensitivity to Chemotherapy

**Figure 9:**
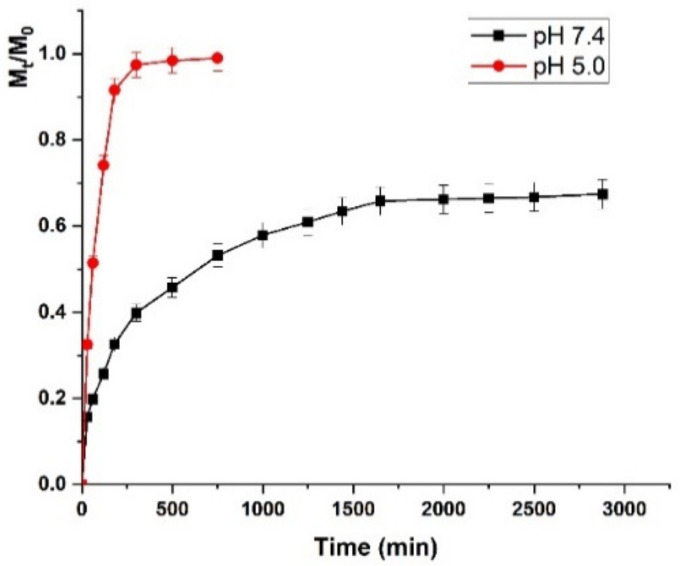
DOXO release profiles from DOXO@ DEX/CUR at pH 5.0 (red line) and pH 7.4 (black lines). Data were reported as mean values ± SD of three independent experiments.

We exploited the self-assembly properties of DEX/CUR to encapsulate DOXO into spherical nanoparticles (DOXO@DEX/CUR, encapsulation efficiency of 85%, loading capacity of 29.5%) and vectorize the chemotherapeutic to PC-3 cancer cells. Initially, the typical DOXO cytotoxicity dose-response was confirmed (Figure S1), which was used to select the drug-to-carrier ratios for subsequent experiments. In detail, we selected two different DOXO concentrations (1.0 and 2.5 µM) to be loaded into DEX/CUR nanoparticles at three different CUR equivalent concentrations (5.0, 10.0, and 20.0 µM), corresponding to a drug-to-carrier ratio ranging from 6.8 to 27.3%. Importantly, as demonstrated in Figure 9, the affinity of DEX/CUR nanoparticles for the chemotherapeutic was found to be strictly dependent on pH.

Here, the release profile clearly demonstrated a better and faster delivery of DOXO at acidic vs. physiological pH, mimicking the characteristics of cancer and normal cells environment, respectively. These results suggest that an effective vectorization of DOXO to cancer cells, with minimal drug release in the surrounding healthy cells, is feasible. More in detail, at pH 7.4, only 60% of the loaded drug was released within 2 days, while at pH 5.0, the release reached 90% in the first 3 h, indicating a lower affinity of DOXO for the DEX/CUR delivery vehicle under these conditions.

**Figure 10:**
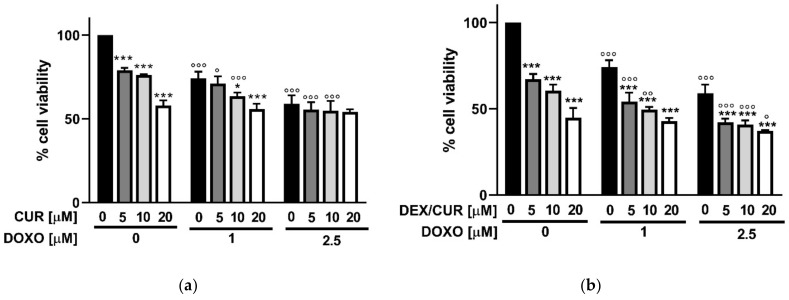
Cell viability assay on PC-3 cells treated for 48 h with a combination of DOXO and (**a**) CUR and (**b**) DEX/CUR at different concentrations. Data were reported as mean values ± SD of three independent experiments. Two-way ANOVA followed by Dunnett’s method: * *p* <0.05, and *** *p* < 0.0001, compared to DOXO single treatments. ° *p* < 0.01, °° *p* < 0.001, °°° *p* < 0.0001 vs. CUR or DEX/CUR single treatments.

The effect of the different nanoformulations on PC-3 viability was assessed, as reported in Figure 10. A dose-dependent cytotoxic effect was recorded in most cases. In detail, when considering the 2.5 μM DOXO concentrations, it is evident that the combination with free CUR did not significantly affect the cell viability compared to the DOXO single treatments (Figure 10a), while the loading into DEX/CUR nanoparticles (Figure 10b) increased the sensitivity of prostate cancer cells to DOXO treatment, thus suggesting the possibility of reducing the doses of chemotherapy used in therapy.

**Figure 11:**
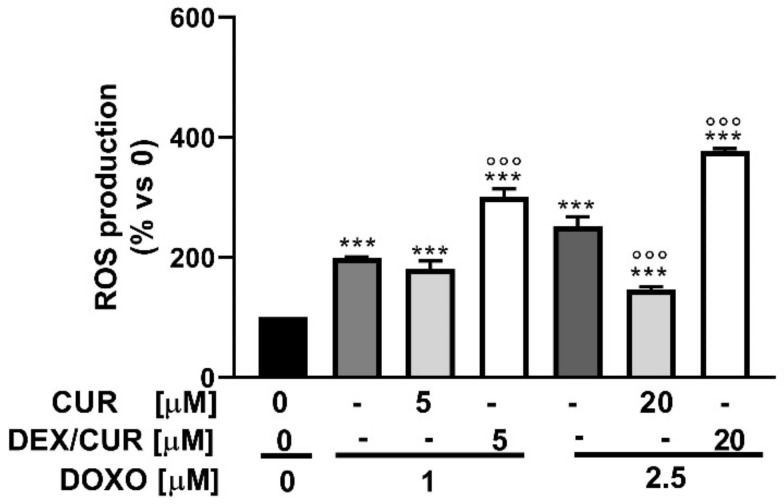
ROS levels (expressed as % of ROS production compared to the control) determined by FACS analysis using the CM-H2DCFDA probe in PC-3 cells after treatment with DOXO (1.0 and 2.5 µM) in combination with CUR and DEX/CUR (5 and 20 µM CUR equivalent concentration). Data were reported as mean values ± SD of three independent experiments. Two-way ANOVA followed by Dunnett’s method: *** *p* < 0.0001, compared to DOXO single treatments. °°° *p* < 0.0001 vs. CUR or DEX/CUR single treatments.

The lower (1.0 µM DOXO + 5 µM CUR/DEX/CUR) and higher (2.5 µM DOXO + 20 µM CUR/DEX/CUR) combination concentrations were tested in a H2DCFDA assay (Figure 11).

The results demonstrated that DOXO induces ROS production in a concentration-dependent manner, with values reaching 198% and 252% of the control at 1.0 and 2.5 µM, respectively. As expected, the combination of DOXO at low dose (1.0 µM) with CUR or DEX/CUR (5.0 µM) resulted in improved ROS production, consistent with the synergistic activity observed in the viability assay. On the other hand, the higher combination of DOXO (2.5 µM) and CUR (20 µM) was ineffective in enhancing ROS production, as anticipated by the antagonistic effect observed in the cell viability assays. Interestingly, the combination treatment with DEX/CUR nanoparticles demonstrated an additive effect on ROS production, with ROS values (%) being exactly the sum of DEX/CUR and DOXO contributions, while the viability assays suggested an antagonistic effect.

## 3. Discussion

With the aim of investigating a Doxorubicin (DOXO) combination therapy for the effective treatment of prostate cancer, we used previously developed, self-assembling nanoparticles based on a DEX/CUR conjugate [40].

DOXO is one of the most widely used and effective therapeutic drugs for a broad number of epithelial cancers, including prostate cancer [44,45,46].

The choice of CUR as bioactive element of the functional nanocarrier arises from the well-known anticancer properties of the polyphenols which were extensively investigated in many studies reporting its ability to modulate different signaling pathways involved in tumor progression [47,48,49]. Furthermore, CUR nanoformulations have been proven to overcome the poor water solubility and potency of CUR hindering its applicability in clinical protocols [50,51].

On the other hand, dextran is a biocompatible polysaccharide, widely proposed for the preparation of highly effective nanoparticle systems [52,53,54]. Interestingly, literature data clearly describe its inability to interfere with any cellular pathway involved in cancer progression and invasion, including EMT [55,56]. For this reason, it is used for the synthesis of fluorescence dyes of biological interest [57,58,59].

The conjugation of CUR to DEX resulted in an improved anticancer activity, with estimated IC_50_ values (expressed as CUR equivalent concentration) decreasing from 27.24 µM for the free form of the polyphenol to 12.21 µM in the case of the bioconjugate. This behavior is consistent with the trend previously recorded for breast cancer cells, and with literature data proving that the anticancer efficiency of polyphenols can be significantly improved by conjugation with polymeric materials undergoing fast cell uptake [60,61]. Moreover, the results of immunofluorescence experiments, together with the cell viability assay on healthy prostate PNT-2 cells, suggest that DEX/CUR can act as highly effective intracellular delivery vehicle able to improve anticancer activity without the simultaneous insurgence of toxic side effects.

After this preliminary characterization, we extensively investigated the mechanism by which DEX/CUR conjugates promote cell death in PC-3 cells, considering that literature data suggest that the cytotoxic effect of CUR is associated with cell cycle arrest, apoptosis and increase in reactive oxygen species (ROS) production [62,63,64]. We proved a G2/M phase arrest and that the decrease in cell growth was associated with apoptosis (Figure 4 and Figure 5) and reactive oxygen species (ROS) generation (Figure 6). ROS are mediators of apoptosis, because of their reactivity towards several macromolecules involved in the electrochemical mitochondrial membrane balance [65,66], and their intracellular level is strictly regulated [67]. The enhanced ROS production upon treatment with DEX/CUR is in agreement with the literature, where several publications have shown the pro-oxidant/anti-oxidant paradox of polyphenols. This translates in an antioxidant effect in the presence of low oxidative stress and a pro-oxidant effect under high oxidative conditions [68,69]. The potential suitability of DEX/CUR for biomedical applications is further demonstrated by the results of ROS production in healthy cells (Figure 6b), since it was found to be safe for normal cells, while exploiting the lack of balance of ROS in cancer cells to selectively induce ROS production, cell death, as well as deregulation of proteins involved in tumorigenesis.

The latter statement was proved by western blot and qRT-PCR analyses, suggesting that the reduction in ZEB-1 expression by CUR (and even more by DEX/CUR) may induce a mesenchymal-to-epithelial transition (MET). This is of great importance, because allowed hypothesizing a lower ability to metastasize, according to the behavior of tumors derived from epithelial tissues accounting for most human cancers, including prostate. After their initial transformation, epithelial cancer cells are confined to the primary site by the continued expression of adhesion molecules and by the intact basal lamina. As the cancer progresses, some cells on the periphery of the primary tumor undergo the epithelial-mesenchymal transition program (EMT), thus acquiring mesenchymal properties, which facilitate the invasion into local and distant tissues (metastases) [70].

Finally, DEX/CUR nanosystem was tested as DOXO delivery vehicle, investigating either the release profile and the result of combination treatments.

The release profiles were analyzed with two mathematical models describing the first order (Equation (1)) and diffusion (Equation (2)) kinetics in order to better understand this behavior. The models are represented by the following Equations:

(1) $$\frac{M_{t}}{M_{0}}=a\left(1-e^{-K_{1}t}\right)$$

In (1) *K*_1_ is the first order kinetic constant while t is the time of the release and *a* is the release coefficient.

(2) $$\frac{M_{t}}{M_{0}}=K_{F}t^{1/2}+K_{a}t$$

In (2) *K_F_* and *K_a_* are the kinetic constants of Fickian and anomalous diffusion, respectively. From the kinetics parameters summarized in Table 1, it is evident that the release is better described (higher R^2^ values) by a first order kinetics at pH 5.0, indicating lower interaction between the cytotoxic drug and the DEX/CUR nanoparticles.

Conversely, diffusion phenomena occurred at pH 7.4 because of stronger drug-to-carrier affinity. Moreover, both models showed that decreased pH significantly enhanced the kinetic constants, supporting the fast release recorded under acidic conditions.

To determine the actual anticancer effect at each tested concentration of DOXO in combination with CUR or encapsulated into DEX/CUR, we introduced Equation (3) [71]:

(3) $$CI=\frac{D_{1}}{D_{x1}}+\frac{D_{2}}{D_{x2}}$$

In (3) *D*_1_ and *D*_2_ are the respective doses of drug 1 and drug 2 in the combination regimen, while *D_x_*_1_ and *D_x_*_2_ are the doses of the single agents (drug 1 or drug 2) that would give the same effect as the combination (*D*_1_ + *D*_2_). *D_x_*_1_ and *D_x_*_2_ values were estimated from the dose-effect data of the single drug treatments.

A CI < 1 means synergism, CI = 1 additive effect, and CI > 1 antagonism. The calculated CI values (Table 2) demonstrated that the loading of DOXO into DEX/CUR nanoparticles resulted into a synergistic effect at low DOXO (1.0) and CUR equivalent (5, 10) concentrations.

Importantly, a slight additive effect was recorded when 1.0 µM DOXO was loaded into 20 µM DEX/CUR, or when 2.5 µM DOXO was loaded into either 5 or 10 µM DEX/CUR. On the other hand, the 2.5 µM DOXO/20 µM DEX/CUR combination resulted in an antagonistic effect.

Control experiments involving combining DOXO and free CUR resulted in a slight additive effect when 5 and 10 µM CUR were combined with 1.0 or 2.5 µM DOXO, but becoming antagonistic when 20 µM was used. These results further confirm that DEX/CUR nanoparticles can be used as DOXO delivery vehicle, with improved anticancer efficiency due to the cytotoxic effect of CUR conjugation to the DEX backbone, or the fast cell uptake of the DOXO@DEX/CUR nanosystem.

Since the combination of DOXO and CUR involved the encapsulation of the chemotherapeutic within the nanoparticles, this may have resulted in a different availability of the chemotherapeutic within the cell environment and thus in a different behavior of CUR and DEX/CUR in DOXO combination treatment.

Although more experiments are needed to further investigate the development of antagonistic effect upon increase of DEX/CUR and DOXO concentration, the results of this study can be considered as a proof-of-principle for the development of combination therapy for the treatment of prostate cancer.

## 4. Materials and Methods

### 4.1. Cell Culture and Treatments

The prostate cancer PC-3 cell line was obtained from American Type Culture Collection (ATCC) and maintained at 37 °C in 5% CO_2_ in culture medium. The normal prostate PNT-2 cell line was kindly provided by Prof. Sisci (University of Calabria, Rende, Italy) and maintained at 37 °C in 5% CO_2_ in culture medium. PC3 and PNT-2 cells were grown in RPMI 1640 medium supplemented with 10% fetal bovine serum (FBS), 1% L-glutamine and 1% penicillin/streptomycin. Cells were cultured at 37 °C for no more than 20 passages. For experiments cells were plated in complete medium, 24 h later treated in medium without serum for the indicated times, as descripted in Results section.

All chemicals were from Sigma/Merck (Darmstadt, Germany).

### 4.2. Synthesis of Doxorubicin Loaded Nanoparticles and Evaluation of Release Profiles

DEX/CUR conjugate and DOXO loaded (DOXO@DEX/CUR) nanoparticle systems were prepared as previously described [40]. For DOXO@DEX/CUR, 2.5 mg DEX/CUR conjugate were added to 5.0 mL DOX solution (0.25 mg × mL^−1^) in PBS under stirring at room temperature for 3 h.

The entrapment efficiency (E.E.) was determined by dialysis method according to the literature [72]. 1.5 mL DOXO loaded DEX/CUR was inserted into a dialysis bag (MWCO: 12,000–14,000 Da), and dialyzed against 25 mL distilled water for 30 min. *EE* (%) was calculated from the ratio of the amount of DOXO entrapped to the total amount of DOXO used in the preparation of nanoparticle, with reference solution being sample not dialyzed, according to the following Equation (4):

(4) $$EE\;\left(\%\right)=\frac{ND-D}{ND}\times 100$$

where *ND* and Dare the drug concentrations before and after the dialysis, respectively.

The drug loading (*DL*) was calculated according to the following Equation (5):

(5) $$DL\;\left(\%\right)=\frac{W_{DOXO}}{W_{DOXO@DEX/CUR}}\times 100$$

The release experiments were performed in phosphate buffered saline (1.0 mM) at pH 7.4 or acetate buffer (1.0 mM) at pH 5.0. Briefly, 1.5 mL DOXO loaded DEX/CUR into phosphate buffered saline at selected pH into a dialysis bag (MWCO: 12,000–14,000 Da), and dialyzed against 13.5 mL of the corresponding buffer. At predetermined time intervals, the amount of DOX in the releasing media was determined by UV–Vis analysis on an Evolution 201 spectrophotometer (ThermoFisher Scientific, Hillsboro, OR, USA) operating with 1.0 cm quartz cells at 496 nm. From the calibration curves of DOX sketched in the same buffer, the cumulative amount of release was calculated using Equation (6):

(6) $$\frac{M_{t}}{M_{0}}=\frac{M_{t}}{M_{total}}$$

where *M_t_* and *M*_0_ are the amounts of drug in solution at time t and loaded into the carrier, respectively.

All chemicals were from Sigma/Merck (Darmstadt, Germany).

### 4.3. Cell Viability Assay

Cell proliferation was evaluated by using the MTT assay. Briefly, PC-3 and PNT-2 cells were seeded at a density of 5 × 10^4^ cells/well (0.5 mL/well) in 24-well plates (Corning Inc. Tewksbury, MA, USA). After 24 h, cells were exposed to different concentrations (0.5, 1, 2, 5, 10 and 20 μM) of curcumin (CUR) or dextran/curcumin (DEX/CUR), for 24 and 48 h, and/or to different concentrations of doxorubicin (DOXO) and DOXO@DEX/CUR for 24 and 48 h. After the exposure treatment, 0.5 mg mL^−1^ of MTT solution was added to each well and the cells were incubated in the dark for an additional 4 h. Then, 200 μL of DMSO was added to each well and the plates were agitated for 10 min at room temperature. The absorbance of the samples was measured at 570 nm using a microplate reader (Synergy H1, BioTek Inc. Winooski, VT, USA). The optical density (*OD*) was calculated as the difference between the absorbance at the reference wavelength and that at the test wavelength. Percent viability was calculated according to the following Equation (7):

(7) $$Viability\;\left(\%\right)=\frac{OD_{sample}}{OD_{control}}\times 100$$

All chemicals were from Sigma/Merck (Darmstadt, Germany).

### 4.4. Staining for Confocal Microscopy

Using the intrinsic fluorescence property of curcumin, the cellular uptake of the drug was visualized in PC-3 cells using a confocal laser scanning microscope (Fluoview FV300 confocal laser scanning microscope, Olympus Corporation, London, UK). To this end, the cells were grown overnight on coverslips, washed in PBS and incubated with vehicle alone or with 20 μM of CUR or DEX/CUR for 4 h at 37 °C. After tree washes in PBS, cells were fixed in 4% paraformaldehyde for 15 min at room temperature and permeabilized in 0.5% triton X-100 in PBS supplemented with 3% BSA (bovine serum albumin) for 10 min at room temperature. Cells were next incubated with RNasi 100 μg mL^−1^ for 20 min at 37 °C followed by incubation with 500 nM of Propidium Iodide (PI) for 5 min at room temperature to selectively label DNA. Finally, cells were washed three times with PBS and the coverslips mounted with Aquapolymount antifading solution on glass slides and observed under the confocal microscope using a 40× objective.

All chemicals were from Sigma/Merck (Darmstadt, Germany).

### 4.5. Cell Cycle Analysis

In order to assess the cell cycle, PC-3 cells were seeded in well plates, at a density of 2.5 × 10^5^ cells/well, and treated with vehicle alone or with 20 μM of CUR or DEX/CUR for 24 h. Flow cytometry was performed as described in [73]. Briefly, PC-3 cells were harvested after treatment with 0.025% trypsin for 3 min at 37 °C and then, after addition of 10% FBS in PBS, the cells were centrifuged, washed with PBS, and fixed in 70% cold ethanol. The harvesting of cells using these conditions led to high yield of undamaged cells. After fixation, cells were washed with PBS, treated for 15 min at 37 °C with RNase (100 μg mL^−1^) and then stained with PI (10 μg mL^−1^ in the dark for 30 min). The samples were then analyzed using a CytoFLEX Beckman flow cytometer (Beckman Coulter Inc., Brea, CA, USA).

All chemicals were from Sigma/Merck (Darmstadt, Germany).

### 4.6. Comet Assay

To measure DNA damage, PC-3 cells were first plated in growth medium for 24 h in 6-well plates at a density of 1 × 10^5^ cells/well, then treated with vehicle alone or with 20 μM of CUR or DEX/CUR for 36 h. Glass slides were coated in 1% agarose and left at 4 °C overnight. After 36 h of incubation, a mixture of 100 µL of cells and 100 µL of 1% agarose was added rapidly on previously prepared slides and covered with coverslips and allowed to solidify for 10 min in ice. Coverslips were gently removed and slides were then placed in a tank with lysis buffer (2.5 M NaCl, 0.1 M EDTA, 10 mM Trizma, 200 mM NaOH, 0.5% Triton X-100, 10% DMSO, pH 10) at 4 °C for 1 h in the dark, and then incubated in alkaline buffer (300 mM NaOH, 1 mM EDTA, pH > 13) at room temperature for 15 min to allow the DNA to unwind. Electrophoresis was performed at room temperature in ice-cold alkaline electrophoresis buffer for 1 h at 40 V. After electrophoresis, cells were fixed in 70% ethanol for 15 min and stained with 30 µL of DAPI solution (0.2 µg × mL^−1^) and then covered with coverslips. Slides were visualized using a 20× objective on a fluorescent microscope (Fluoview FV300 confocal laser scanning microscope, Olympus Corporation).

All chemicals were from Sigma/Merck (Darmstadt, Germany).

### 4.7. ROS Detection Assay

The production of the reactive oxygen species (ROS) in cells was detected by using the general oxidative stress indicator (CM-H2DCFDA, Invitrogen, Carlsbad, CA, USA). PC-3 and PNT-2 cells were plated and left in growth medium for 24 h, and then treated for 24 or 48 h with CUR (or DEX/CUR), DOXO alone or with DOXO@DEX/CUR. Cells were collected and the pellet was incubated in a mix containing PBS and 5 µM of the dye chloromethyl-2′,7′-dichlorofluorescien diacetate (CM-H2DCFDA) at 37 °C with 5% of CO_2_. After 1 h, cells were incubated with growth medium at 37 °C for 20 min. The assay allows to quantify total intracellular ROS thanks to the entry of DCFHDA into cell membranes and the subsequent enzymatic hydrolysis of intracellular esterases into nonfluorescent DCFH [74]. In the presence of ROS, DCFH is rapidly oxidized to highly fluorescent 2′,7′-dichlorofluorescein (DCF), detectable by a fluorescence microplate reader in an excitation/emission range of 492–495/517–527 nm. In each sample, probe fluorescence is normalized on viable cells, counted by means of Trypan Blue using a 10× objective on a microscope (CKX31 Olympus).

Unless otherwise indicated, all chemicals were from Sigma/Merck (Darmstadt, Germany).

### 4.8. Western Blotting Analysis

PC3 cells were treated with vehicle or with 20 μM of CUR (or DEX/CUR) for 48 h. Then proteins were extracted using RIPA buffer containing cocktail inhibitors (Sigma/Merck, Darmstadt, Germany) and concentration was determined using a Bradford dye-based assay (Bio-Rad, Milan, Italy). Total protein (30 μg of protein/lane) was subjected to SDS–PAGE followed by immunoblotting with appropriate antibodies at the recommended dilutions. The blots were then incubated with peroxidase linked secondary antibodies followed by enhanced-chemiluminescent detection using Super Signal chemiluminescence kit (Thermo Fisher Scientific, Waltham, MA, USA). The following antibodies were used: ZEB-1 (1:500; Santa Cruz Biotehcnology, Dallas, TX, USA), E-cadherin (1:1000; Cell Signaling, Leiden, The Netherlands), vimentin (1:1000; Cell Signaling, Leiden, The Netherlands), GAPDH (1:1000; Santa Cruz Biotehcnology, Dallas, TX, USA).

### 4.9. RNA Extraction, cDNA Synthesis and qRT-PCR

Total RNA was isolated from cells using Trizol according to the manufacturer’s instructions. RNA samples were treated with RNase-free DNase I. Total RNA was reverse transcribed using the OneScript Plus cDNA Synthesis Kit and oligo (dT) primer (ABM) (Thermo Fisher Scientific, Waltham, MA, USA). qRT-PCR was performed using an ABI PRISM 7000 Sequence Detection System with SYBR green ready mix (Applied Biosystem, Thermo Fisher Scientific, Waltham, MA, USA) and specific primers (see below). GAPDH gene was used as an internal control. Gene expression was defined from the threshold cycle (Ct), and relative expression levels were calculated using the 2^−ΔΔCt^ method after normalization with reference to expression of housekeeping gene (GAPDH)

Primers for qRT-PCR:

Human ZEB1 for GCACCTGAAGAGGACCAGAG

Human ZEB1 rev TGCATCTGGTGTTCCATTTT

Human E-cadherin for CCCACCACGTACAAGGGTC

Human E-cadherin rev CTGGGGTATTGGGGGCATC

Human Vimentin for GACAATGCGTCTCTGGCACGTCTT

Human Vimentin rev TCCTCCGCCTCCTGCAGGTTCTT

Human GAPDH for AGCCACATCGCTCAGACAC

Human GAPDH rev GCCCAATACGACCAAATCC

All reagents were from Invitrogen, Carlsbad, CA, USA.

### 4.10. Wound Healing Assay

PC3 cells were plated in six-well plates, treated with vehicle or with 20 μM of CUR (or DEX/CUR), and kept in culture. After 24 h, cells were scraped with a p200 tip (time 0 h), washed several times in PBS, and monitored with a phase contrast 10× objective using an Olympus BX41 microscope with CSV1.14 software and a CAMXC-30 for image acquisition. Images were obtained immediately (0 h) and 24 h after scratching, and were representative of the three independent experiments.

### 4.11. Statistical Analysis

Data were reported as mean values ± SD of three independent experiments. One-way or two-way analysis of variance (ANOVA) followed by Dunnett’s method were used to generate statistical analyses, using the GraphPad Prism software. *p* values < 0.05 were considered statistically significant.

## 5. Conclusions

We presented experimental evidence that DEX/CUR nanoparticles can act as a nanoplatform to provide a pH-responsive release of the cytotoxic agent DOXO, and enhance its anticancer activity. Nanoparticles were obtained by a straightforward procedure involving the self-assembly of a DEX/CUR conjugate in water media. The biological characterization of the nanoparticles, including improved toxicity toward cancer cells and safety for normal cells, the induction of ROS production and apoptosis, as well as the inhibition of cell migration, confirmed their suitability as functional delivery vehicles. Moreover, the pH responsive release profile, together with the evaluation of the combination index, clearly demonstrated the ability of such nanoparticles to vectorize the drug to the tumor site while synergizing its anticancer effect, indicating the development of a valuable tool for the treatment of prostate cancer. Although in vivo experiments are required to prove the actual therapeutic performance of the proposed nanosystem, the reported preliminary in vitro data can be considered a proof of principle for the design of effective vehicles for prostate cancer treatment.

## Figures and Tables

**Table 1:** Kinetic parameters for DOXO release profiles from DOXO@DEX/CUR at pH 5.0 and 7.4.

Model	R^2^		*K*	
	pH 5.0	pH 7.4	pH 5.0	pH 7.4
Equation (1)	0.9978	0.5855	0.0122	0.0007
Equation (2)	0.9575	0.9985	0.0868 ^1^ 0.0019 ^2^	0.0271 ^1^ 0.0003 ^2^

^1^*K_F_*; ^2^*K_a_*.

**Table 2:** Combination index for CUR/DOXO and DOXO@DEX/CUR combination treatments.

Treatment	[µM] ^1^	DOXO [µM]	
		1.0	2.5
CUR	5	0.91	1.04
	10	1.02	1.17
	20	1.35	1.48
DEX/CUR	5	0.72	0.90
	10	0.81	0.95
	20	0.95	1.31

^1^ CUR equivalent concentration.

## Data Availability

Not applicable.

## Supplementary Materials

The following are available online at https://www.mdpi.com/article/10.3390/ijms22137013/s1, Figure S1: PC-3 viability after treatment with DOXO.
