# Supplementary material for: Dextran-Curcumin Nanosystems Inhibit Cell Growth and Migration Regulating the Epithelial to Mesenchymal Transition in Prostate Cancer Cells
Source: Int J Mol Sci. 2021 Jun 29;22(13):7013. doi: 10.3390/ijms22137013 (PMC8269310; doi:10.3390/ijms22137013)
Supplement: Supplementary file 1 [file ijms-22-07013-s001.zip › ijms-1248139-supplementary.pdf]

International Journal of *Molecular Sciences*

Supplementary Materials
Dextran-Curcumin nanosystems inhibit cell growth and migration regulating the epithelial to mesenchymal transition in prostate cancer cells

Emilia Bevacqua [1], Manuela Curcio [1], Federica Saletta [2,3], Orazio Vittorio [2,3,4], Giuseppe Cirillo [1] and Paola Tucci [1,*]

[1] Department of Pharmacy, Health and Nutritional Sciences, University of Calabria, Rende (CS) 87036, Italy; emilia.bevacqua@unical.it (E.B.); manuela.curcio@unical.it (M.C.); giuseppe.cirillo@unical.it (G.C.)
[2] Children's Cancer Institute, Lowy Cancer Research Centre, UNSW Sydney, NSW 2031, Australia; OVittorio@ccia.unsw.edu.au (O.V.); FSaletta@ccia.org.au (F.S.)
[3] School of Women's and Children's Health, Faculty of Medicine, UNSW Sydney, NSW 2052, Australia
[4] ARC Centre of Excellence for Convergent BioNano Science and Technology, Australian Centre for NanoMedicine, UNSW Sydney, NSW 2052, Australia
* Correspondence: paola.tucci@unical.it; Tel.: 0039 0984493185

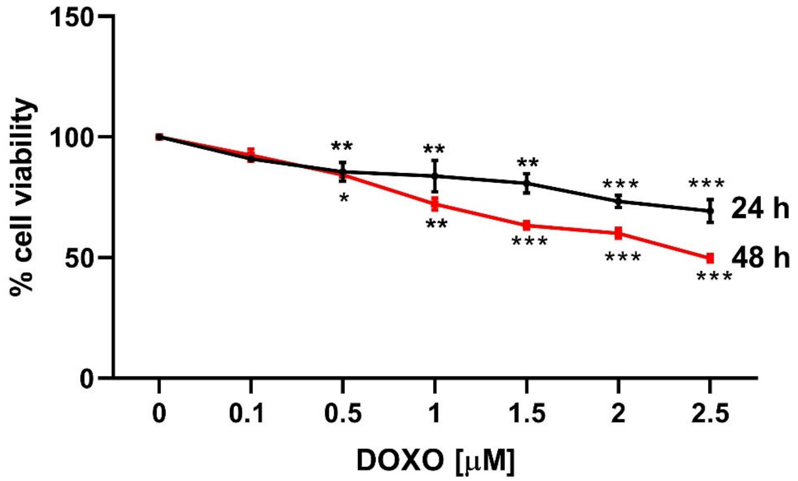

Figure S1. PC-3 viability after 24 h (Black line) and 48 h (red line) treatment with DOXO. *$p < 0.01$, **$p < 0.001$, ***$p < 0.0001$ vs control.
